# COMMON UNDERLYING FACTORS FOR KNOWN MODIFIABLE FALL RISKS

**DOI:** 10.1093/geroni/igz038.2092

**Published:** 2019-11-08

**Authors:** Gwen Bergen, Ramakrishna Kakara, Elizabeth Burns, Mark R Stevens

**Affiliations:** 1 Centers for Disease Control and Prevention, National Center for Injury Prevention and Control, Division of Unintentional Injury Prevention, Atlanta, Georgia, United States; 2 Division of Unintentional Injury, National Center for Injury Prevention and Control, Oak Ridge Institute for Science and Educaton, Atlanta, Georgia, United States; 3 Centers for Disease Control and Prevention, Atlanta, Georgia, United States

## Abstract

Certain demographics, health conditions, and functional limitations are associated with increased older adult falls. Health conditions and functional limitations are potentially modifiable and may have underlying factors in common. This study’s objective is to understand whether health conditions and functional limitations related to increased fall risk have common underlying factors that could be useful in designing interventions. Factor analysis and multivariate logistic regression were used to analyze 2016 Behavioral Risk Factor Surveillance Survey data for adults aged 65+ years. About half of those who reported difficulty dressing (58%), difficulty running errands alone (53%), difficulty remembering (51%) and depression (48%) reported falling compared to 30% of the general older adult population. Two common factors of cognitive and physical limitations were identified and scales created for each. When controlling for demographic characteristics, both cognitive and physical limitations scales were significantly related to a higher odds of falling (Odds ratios=1.5, 1.4 respectively).

